# TNFAIP3 genetic polymorphisms reduce ankylosing spondylitis risk in Eastern Chinese Han population

**DOI:** 10.1038/s41598-019-46647-1

**Published:** 2019-07-15

**Authors:** Jiajia Yang, Xingxing Hu, Meng Wu, Yubo Ma, Xu Zhang, Mengya Chen, Yaping Yuan, Renfang Han, Rui Liu, Shiyang Guan, Jixiang Deng, Shanshan Xu, Xing Gao, Shengqian Xu, Zongwen Shuai, Shanqun Jiang, Shihe Guan, Liwen Chen, Faming Pan

**Affiliations:** 1 Department of Epidemiology and Biostatistics, School of Public Health, Anhui Medical University, 81 Meishan Road, Hefei, Anhui China; 2 The Key Laboratory of Major Autoimmune Diseases, Anhui Medical University, 81 Meishan Road, Hefei, Anhui China; 3 Department of Rheumatism and Immunity, the First Affiliated Hospital of Anhui Medical University, Hefei, Anhui China; 4 School of Life Sciences, Anhui University, 111 Jiulong Road, Hefei, Anhui China; 5 Department of Clinical Laboratory, the Second Hospital of Anhui Medical University, 678 Furong Road, Hefei, Anhui China

**Keywords:** Biological sciences, Genetics, Genetic association study

## Abstract

This study was conducted to clarify the associations of tumor necrosis factor-α induced protein 3 (TNFAIP3) and TNFAIP3-interacting protein 1 (TNIP1) genetic polymorphisms with ankylosing spondylitis (AS) susceptibility. Five single nucleotide polymorphisms (SNPs) in TNFAIP3 gene and four in TNIP1 gene were genotyped in 667 AS patients and 667 matched healthy controls. Genotypes and haplotype analysis were conducted by using SPSS 23.0 and Haploview 4.2 software. The T allele and CT genotype in TNFAIP3 rs10499194 were significantly associated with a reduced AS risk (T allele vs. C allele, OR = 0.619, 95% CI = 0.430–0.889, *P* = 0.009; CT vs. CC, OR = 0.603, 95% CI = 0.416–0.875, *P* = 0.007). However, no association remained significant after Bonferroni correction. The rs13207033^A^- rs10499194^T^ haplotype of TNFAIP3 conferred a protective effect on AS susceptibility. Stratification analyses suggested that rs10499194 polymorphism decreased the risk of AS in the male subgroup, subgroup aged ≥ 29, HLA-B27 positive subgroup as well as the subgroups of BASFI < 4 and BASDAI < 4 (all *P* < 0.05). Furthermore, the functional annotation suggested a potential function of rs10499194 mutation. Our results demonstrated that TNFAIP3 rs10499194 polymorphism may be associated with a reduced risk of AS.

## Introduction

Ankylosing spondylitis (AS), as a chronic inflammatory autoimmune disease, mainly involves axial skeleton and sacroiliac joints, resulting in impairments of structure and function^1,2^. The estimated prevalence of AS among the global population is 0.7–3.2%^3^, and the number of male patients is approximately twice that of female patients^4^. At present, although the nosogenesis of AS remains unclear, genetic predisposition, innate immune aspects combined with environmental factors were recognized to exert a crucial role in the development of AS^5,6^. Previous studies have confirmed that the major histocompatibility complex (MHC) haplotype human leukocyte antigen B27 (HLA-B27) is associated with the etiology of AS, however, HLA-B27 only accounts for 20%–30% of the genetic susceptibility to AS^1^. Up to now, many potential non-HLA AS-associated genetic factors including some susceptibility loci have been discovered in genome-wide association study (GWAS)^7,8^. Nevertheless, because the associations having been found cannot completely explain this disease, there are undoubtedly other genetic aspects implicated in AS pathogenesis.

Tumor necrosis factor (TNF)-α induced protein 3 (TNFAIP3) gene encodes a ubiquitin-modifying enzyme which is known as A20. A20 is a crucial negative regulatory factor of nuclear factor- kappaB (NF-κB) signal pathway. The over-activation of NF-κB may give over-expressions to many pro-inflammatory genes, thus triggering inflammatory responses and tissue lesions^9,10^. A20 participates in mediating immune and inflammatory responses by suppressing the function of NF-κB. Furthermore, plenty of evidence showed that A20 could restrict B cell survival and prevent autoimmune pathology^11,12^. Meanwhile, A20 was found to play a significant role in regulating the maturation and function of dendritic cells (DCs). A20-silenced DCs inhibited regulatory T cells (Tregs), T helper (Th) cells as well as hyperactivated tumor-infiltrating cytotoxic T lymphocytes that produced interleukin-6 (IL-6) and TNF-α^13^. As is known, A20 binds to A20-binding inhibitor of NF-kB activation (ABIN1), a polyubiquitin-binding protein encoded by the TNFAIP3-interacting protein 1 (TNIP1) gene^14^. In addition to enhancing the NF-κB inhibitory function of A20, TNIP1 could compete with NF-κB mediator molecules for polyubiquitin, thus restraining the process of ubiquitination and subsequent NF-κB activation^15^. The establishment of TNIP1-deficient mice indicated that TNIP1 knockout could cause multiple inflammatory symptoms with several characteristics of progressive lupus-like disease like leukocyte infiltrations, T and B cell excitation and autoantibody production^16^.

Recently, numerous researches have revealed that TNFAIP3 or TNIP1 gene polymorphisms were associated with susceptibility to some autoimmune inflammatory diseases including systemic lupus erythematous (SLE), systemic sclerosis (SSc), rheumatoid arthritis (RA) and psoriasis^17–21^. Nevertheless, there is hardly any study about investigating the associations between single nucleotide polymorphisms (SNPs) from TNFAIP3 and TNIP1 genes and AS risk. Hence, we implemented this case-control study to determine the relationships of five TNFAIP3 polymorphisms (rs610604, rs10499194, rs13207033, rs2230926 and rs6920220) and four TNIP1 polymorphisms (rs2233287, rs3792783, rs4958881 and rs6889239) with AS susceptibility.

## Results

### Demographic and clinical characteristics

Six hundred and sixty-seven patients with AS (542 males, 125 females) and 667 healthy controls (559 males, 108 females) were included in the present study. The mean age of patients and controls was 28.47 ± 9.08 and 28.88 ± 7.68 years, respectively. There were no statistical differences between AS cases and healthy controls in terms of gender and age. Among all AS patients, 412 (61.78%) were positive for HLA-B27. The other specific clinical characteristics of AS subjects are demonstrated in Table 1.

**Table 1:** Demographic and clinical characteristics of participants.

	AS (n = 667)	HC (n = 667)	*P*
Gender (male/female)	542/125	559/108	0.220
Age (years, mean ± SD)	28.47 ± 9.08	28.88 ± 7.68	0.369
BMI (kg/m 2, mean ± SD)	22.23 ± 3.97		
Age at presentation(years, mean ± SD)	23.60 ± 9.77		
Disease course (year, median (IQR))	3.00 (1.00, 8.00)		
HLA-B27 positivity (%)	412 (61.78%)		
ESR (mm/h, median(IQR))	15.00 (5.00, 33.00)		
CRP (mg/L, median (IQR))	9.89 (2.64, 29.92)		
WBC (/L, median (IQR))	7.08 (5.86, 8.40)		
BASDAI (score, median (IQR))	2.00 (0.60, 3.80)		
BASFI (score, median (IQR))	0.90 (0.00, 2.60)		

AS, ankylosing spondylitis; HC, healthy controls; BMI, body mass index; ESR, erythrocyte sedimentation rate; CRP, C-reactive protein; WBC, white blood cell; BASDAI, Bath Ankylosing Spondylitis Disease Activity Index; BASFI, Bath Ankylosing Spondylitis Functional Index.

### Genotype distributions, allele frequencies and inheritance models

In control group, these nine polymorphisms were tested for Hardy-Weinberg equilibrium (HWE), and none of them significantly deviate from HWE (all *P* > 0.10). The distribution of genotype and allele frequencies of all SNPs in AS patients and normal controls are summarized in Table 2. There were statistically significant differences between AS and control groups in the genotype distribution and allele frequency at TNFAIP3 rs10499194 (genotype distributions: OR = 0.603, 95% CI = 0.416–0.875, *P* = 0.007; allele frequency: OR = 0.619, 95%CI = 0.430–0.889, *P* = 0.009). However, no association remained statistically significant after Bonferroni correction.

**Table 2:** The allele and genotype frequencies of TNFAIP3 and TNIP1 genes polymorphisms

SNPs	Genotype					Allele						*P*_HWE_
		AS (n, %)	HC (n, %)	*P*	*P*_*C*_		AS (n, %)	HC (n, %)	OR (95% CI)	*P*	*P*_*C*_	
**TNFAIP3**												
rs610604	TT	553 (82.9)	553 (82.9)	0.859	NS	T	1214 (91.0)	1212 (90.9)	(Reference)			0.259
	GT	108 (16.2)	106 (15.9)			G	120 (9.0)	122 (9.1)	0.982 (0.754–1.279)	0.893	NS	
	GG	6 (0.9)	8 (1.2)									
rs10499194	CC	617 (92.5)	588 (88.2)	0.007^a^	NS	C	1284 (96.3)	1255 (94.1)	(Reference)			0.104
	CT	50 (7.5)	79 (11.8)			T	50 (37.5)	79 (5.9)	0.619 (0.430–0.889)	0.009^a^	NS	
	TT	0	0									
rs13207033	GG	544 (81.6)	517 (77.5)	0.181	NS	G	1201 (90.0)	1173 (87.9)	(Reference)			0.639
	GA	113 (16.9)	139 (20.8)			A	133 (10.0)	161 (12.1)	0.807 (0.633–1.029)	0.083	NS	
	AA	10 (1.5)	11 (1.7)									
rs2230926	TT	621 (93.1)	614 (92.1)	0.317	NS	T	1288 (96.6)	1279 (95.9)	(Reference)			0.396
	GT	46 (6.9)	51 (7.6)			G	46 (3.4)	55 (4.1)	0.831 (0.557–1.238)	0.361	NS	
	GG	0	2 (0.3)									
rs6920220	GG	660 (99.0)	662 (99.3)	0.562	NS	G	1327 (99.5)	1329 (99.6)	(Reference)			0.923
	GA	7 (1.0)	5 (0.7)			A	7 (0.5)	5 (0.4)	1.402 (0.444–4.429)	0.563	NS	
	AA	0	0									
TNIP1												
rs2233287	GG	662 (99.3)	667 (100)	0.073	NS	G	1329 (99.6)	1334 (100)	(Reference)			1.000
	GA	5 (0.7)	0			A	5 (0.4)	0	0.996 (0.993–1.000)	0.073	NS	
	AA	0	0									
rs4958881	TT	563 (84.4)	560 (84.0)	0.829	NS	T	1225 (91.8)	1220 (91.5)	(Reference)			0.292
	CT	99 (14.8)	100 (15.0)			C	109 (8.2)	114 (8.5)	0.952 (0.724–1.253)	0.727	NS	
	CC	5 (0.8)	7 (1.0)									
rs3792783	AA	392 (58.8)	398 (59.7)	0.635	NS	A	1030 (77.2)	1030 (77.2)	(Reference)			0.937
	GA	246 (36.9)	234 (35.1)			G	304 (22.8)	304 (22.8)	1.000 (0.835–1.198)	1.000	NS	
	GG	29 (4.3)	35 (5.2)									
rs6889239	CC	382 (57.3)	375 (56.2)	0.902	NS	C	1006 (75.4)	1000 (75.0)	(Reference)			0.969
	CT	242 (36.3)	250 (37.5)			T	328 (24.6)	334 (25.0)	0.976 (0.819–1.164)	0.788	NS	
	TT	43 (6.4)	42 (6.3)									

AS, ankylosing spondylitis; HC, healthy controls; SNPs, single nucleotide polymorphisms; OR, odds ratio; CI, confidence interval; HWE, Hardy-Weinberg equilibrium test; TNFAIP3, tumor necrosis factor-α induced protein 3; TNIP1, TNFAIP3-interacting protein 1; NS, not significant; *Pc*, Bonferroni corrected *P*-value; ^a^*P* < 0.05 after adjusting sex and age.

Dominant, recessive and codominant genetic models in AS patients were compared with that in healthy controls. The results displayed that only the genotype CT at rs10499194 was significantly associated with a decreased risk of AS development when compared with the wild type genotype CC (OR = 0.603, 95%CI = 0.416–0.875; *P* = 0.007). Yet, the *P* value after Bonferroni correction reached no statistical significance (Supplementary Tables S2 and S3). The associations of genotype with clinical characteristics in AS patients were further investigated. But unfortunately, all those polymorphisms were not correlated with the clinical features.

### Association of rs10499194 polymorphism with AS risk in stratification analyses

To further evaluate the association between TNFAIP3 rs10499194 polymorphism and AS risk, stratification analyses by sex, age, HLA-B27 status, BASFI and BASDAI for rs10499194 were performed, and the results are presented in Table 3. The rs10499194 CT genotype decreased the risk of AS when compared to CC genotype in the male subgroup and in the population aged ≥29, and the adjusted ORs were 0.620 (95% CI = 0.416–0.924) and 0.533 (95% CI = 0.292–0.974), respectively. In addition, rs10499194 genotype distribution was different between HLA-B27 positive patients and controls (CT vs. CC, adjusted OR = 0.547, 95% CI = 0.349–0.859, *P* = 0.009), between patients of BASFI < 4 and controls (CT vs. CC, adjusted OR = 0.631, 95% CI = 0.427–0.932, *P* = 0.021), and between patients of BASDAI < 4 and controls (CT vs. CC, adjusted OR = 0.587, 95% CI = 0.391–0.881, *P* = 0.010).

**Table 3:** Stratification analyses by sex, age, HLA-B27 status, BASFI and BASDAI for rs10499194 polymorphism.

Stratum	OR(95% CI)	*P*	Adjusted OR^a^ (95% CI)	*P* ^a^
**Sex**				
Male	0.627 (0.421–0.934)	0.021	0.620 (0.416–0.924)	0.019
Female	0.494 (0.173–1.408)	0.180	0.498 (0.175–1.421)	0.193
**Age**				
<29	0.666 (0.414–1.071)	0.092	0.659 (0.406–1.069)	0.091
≥29	0.517 (0.284–0.941)	0.029	0.533 (0.292–0.974)	0.041
**HLA-B27 status**				
HLA-B27(+)	0.543 (0.346–0.851)	0.007	0.547 (0.349–0.859)	0.009
HLA-B27(−)	0.703 (0.428–1.154)	0.162	0.694 (0.422–1.142)	0.150
**BASDAI**				
<4	0.590 (0.394–0.886)	0.010	0.587 (0.391–0.881)	0.010
≥4	0.647 (0.343–1.221)	0.176	0.667 (0.353–1.261)	0.212
**BASFI**				
<4	0.629 (0.426–0.929)	0.019	0.631 (0.427–0.932)	0.021
≥4	0.482 (0.217–1.073)	0.068	0.482 (0.217–1.073)	0.074

OR, odds ratio; CI, confidence interval; BASDAI, Bath Ankylosing Spondylitis Disease Activity Index; BASFI, Bath Ankylosing Spondylitis Functional Index; ^a^adjusted for sex and age.

### LD and haplotype analysis

**Figure 1:**
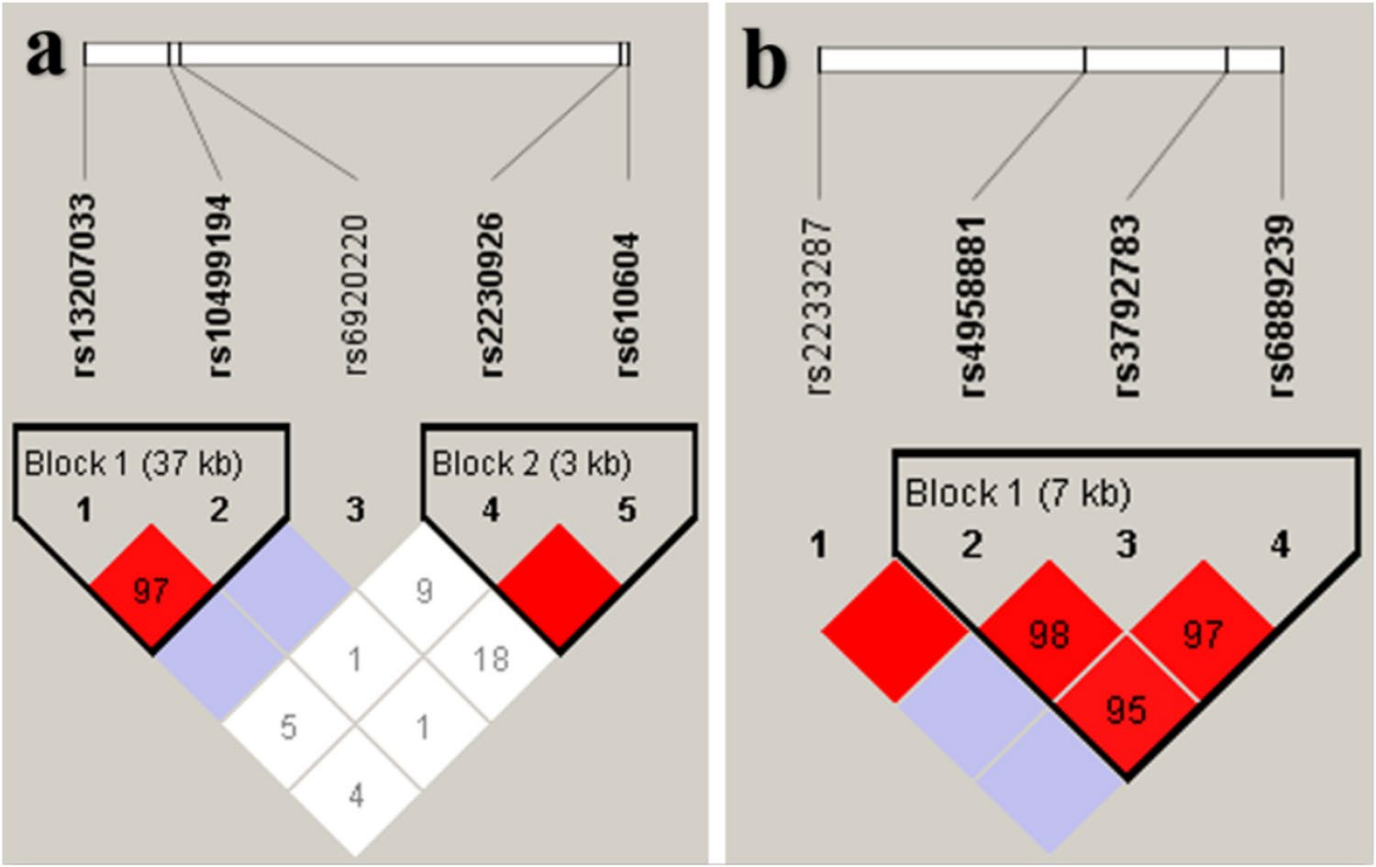
Linkage disequilibrium (LD) analysis of the (**a**) TNFAIP3 and (**b**) TNIP1 SNPs. The LD status is expounded by the D′ value. D′ > 0.9 but *r*^2^ < 0.4 in all the blocks.

LD analysis was performed on the basis of the five SNPs of TNFAIP3 gene and the four SNPs of TNIP1 gene, respectively. For TNFAIP3 gene, the results showed two weak LDs respectively between rs13207033 and rs10499194, and between rs2230926 and rs610604. As for TNIP1 gene, the LD among the SNPs of rs4958881, rs3792783 and rs6889239 was observed, while it was not strong (Fig. 1). Haplotype analysis showed that only the ht3 (rs13207033^A^- rs10499194^T^) haplotype was significantly associated with a reduced risk of AS (OR = 0.601, 95% CI = 0.416–0.868; *P* = 0.006) (Table 4).

**Table 4:** Results of TNFAIP3 and TNIP1 haplotype frequencies.

Combinations of markers	Haplotypes	AS	HC	OR (95% CI)	*P*
**TNFAIP3**					
rs13207033-rs10499194	Ht1 (GC)	0.899	0.878	1.228 (0.964–1.564)	0.096
	Ht2 (AC)	0.064	0.062	1.026 (0.750–1.402)	0.877
	Ht3 (AT)	0.036	0.058	0.601 (0.416–0.868)	0.006
rs2230926-rs610604	Ht4 (TT)	0.876	0.867	1.076 (0.858–1.350)	0.525
	Ht5 (TG)	0.090	0.091	0.982 (0.754–1.279)	0.893
	Ht6 (GT)	0.034	0.041	0.831 (0.557–1.238)	0.361
**TNIP1**					
rs4958881-rs3792783-rs6889239	Ht7 (TAC)	0.528	0.524	1.015 (0.872–1.182)	0.847
	Ht8 (TAT)	0.244	0.248	0.980 (0.822–1.169)	0.823
	Ht9 (TGC)	0.146	0.141	1.044 (0.840–1.296)	0.698
	Ht10 (CGC)	0.081	0.085	0.952 (0.723–1.254)	0.725

AS, ankylosing spondylitis; HC, healthy controls; OR, odds ratio; CI, confidence interval; TNFAIP3, tumor necrosis factor-α induced protein 3; TNIP1, TNFAIP3-interacting protein 1.

### Association of wGRS with AS

Weighted GRS was calculated from the five SNPs in TNFAIP3. The results of Mann-Whitney U test demonstrated that the wGRS was statistically different between AS patients and healthy controls (AS patients: median wGRS = 1.795, IQR = 0.018; healthy controls: median wGRS = 1.795, IQR = 0.185, *Z* = −2.228, *P* = 0.026). The logistic regression analysis of wGRS and AS showed a significant association (OR = 1.936, 95% CI = 1.194–3.138), indicating that with every one point increase in the wGRS, the risk for AS was increased by 1.936. Furthermore, wGRS in HLA-B27 positive patients was different with that in HLA-B27 negative patients (HLA-B27 positive patients: median wGRS = 1.795, IQR = 0.020; HLA-B27 negative patients: median wGRS = 1.795, IQR = 0.188, *Z* = −2.033, *P* = 0.042).

### Investigation of the utility of genetic variants by ROC curve analysis

To examine the clinical utility of the wGRS, ROC curve analysis was applied. The AUC for prediction of AS risk was 0.531 (95% CI = 0.500–0.562; *P* = 0.048), and the optimal point according to the maximum Youden’s index presented a sensitivity of 63.7% and a specificity of 41.2%, suggesting that the wGRS have statistically significant but relatively weak ability to distinguish AS patients from healthy controls (Supplementary Fig. S1).

### Gene-gene interaction with MDR

We performed MDR analysis to explore the possible high-dimension interactions among the SNPs in the study. Case–control status for an individual subject was defined as a binary variable, and SNP variables ranging from 0 to 2 represent the number of mutant alleles in each participant (0 = wild-type homozygote, 1 = heterozygote, 2 = homozygote). However, on testing, there was no statistical interaction effect among all gene loci (Supplementary Table S4).

### Functional variant evaluation

To explore the role of rs10499194 variant in the biological mechanisms, systematic functional annotations were performed for rs10499194 and the SNPs in high LD with it (*r*^2^ > 0.8 in East Asian population). As shown in Table 5, the epigenomics data of primary monocytes and T cells in ROADMAP database demonstrated that rs10499194 and rs77027760 fell within regulation elements marked by H3K4ME3, H3K4ME1 and H3K27AC. Meanwhile, rs10499194 and rs77027760 showed lower scores in Regulome DB (4 for rs10499194 and 3a for rs77027760), indicating that the two SNPs potentially had the relatively abundant function. The two SNPs were further suggested to have the lower *P* values in PINES, suggesting that they might be the functional SNPs. Furthermore, rs10499194 was showed to be in the histone modifications of H3K4me1, H3K4ME3 and H3K27AC in the lymphoblastoid cell line GM12878 retrieved from the UCSC genome browser (Supplementary Fig. S2).

**Table 5:** Comprehensive annotation of SNPs in strong LD (*r*^2^ > 0.8) with rs10499194.

CHR	BP	SNP	*r* ^2^	A1	A2	Gene	Position	H3K4ME3^a^	H3K4ME1^a^	H3K27AC^a^	Regulome DB^b^	Weighted _PINES^c^
6	138002061	rs77027760	0.86	A	G	TNFAIP3	intergenic	T	T	T	3a	0.030
6	138002637	rs10499194	1	T	C	TNFAIP3	intergenic	T	T	T	4	0.184
6	138004508	rs142761146	0.86	A	T	TNFAIP3	intergenic				5	0.851
6	138004568	rs80351603	1	A	G	TNFAIP3	intergenic				6	0.851
6	138005428	rs13205649	1	G	C	TNFAIP3	intergenic				6	0.503
6	138008679	rs66499821	1	C	T	TNFAIP3	intergenic		T		6	0.564
6	138009900	rs34654849	0.91	T	C	TNFAIP3	intergenic		T	T	No Data	0.364
6	138011151	rs12525643	1	G	A	TNFAIP3	intergenic				No Data	0.150

BP: base position; A1: effective allele; A2: reference allele; T: True, own the specific histone modification.

^a^histone modifications were derived from Primary mononuclear and T cells from peripheral blood (Roadmap Epigenomics Consortium, http://www.roadmapepigenomics.org/);

^b^Regulome DB (http://www.regulomedb.org/) annotated SNPs with known and predicted regulatory elements in the intergenic region. The scores refer to the possible regulatory function of a SNP, 3a:TF binding, any motif and DNase peak; 4:TF binding and DNase peak; 5:TF binding or DNase; 6:other function;

^c^PINES (http://genetics.bwh.harvard.edu/pines) provided a powerful in silico method to prioritize and fine map functional noncoding variants. SNPs with lower P values indicated more abundant function.

## Discussion

AS is a complex autoimmune inflammatory disease with strong genetic susceptibility. Investigating the associations between gene polymorphisms and AS is helpful to understand the mechanisms. In the present study, the results indicated that the minor T allele and CT genotype of TNFAIP3 rs10499194 were associated with the decreased AS susceptibility. Nevertheless, no statistical association was observed between the other eight SNPs in TNFAIP3 and TNIP1 genes and AS risk. Through the stratification analyses for rs10499194 genotype distribution, we observed that when compared to CC genotype, the CT genotype decreased the risk of AS in male, aged ≥29, BASFI <4, BASDAI <4 and HLA-B27 positive patients. The rs13207033^A^- rs10499194^T^ haplotype of TNFAIP3 was further revealed to confer protective effect on AS development. In addition, the functional annotation suggested a potential function of rs10499194 mutation.

TNFAIP3 gene encodes the ubiquitin-editing enzyme A20, and TNIP1 gene encode A20-binding inhibitor of NF-kB activation (ABIN1). A20 and ABIN1 are critical regulators for NF-κB signal pathway which is confirmed to be involved in the processes of auto-reactivity and immune activity. Several TNFAIP3 and TNIP1 genetic polymorphisms have been revealed to be associated with various autoimmune disorders including rheumatoid arthritis (RA), systemic lupus erythematosus (SLE), systemic sclerosis (SSc), psoriasis, etc. Hence, we were interested in exploring the associations of SNPs in TNFAIP3 and TNIP1 genes with AS susceptibility, and the five SNPs (rs610604, rs10499194, rs13207033, rs2230926 and rs6920220) in TNFAIP3 gene and four SNPs (rs2233287, rs4958881, rs3792783 and rs6889239) in TNIP1 gene were selected. It has been reported that TNFAIP3 rs610604 was genetic susceptibility loci to psoriasis^19,22^ and Graves’ disease^23^, and the rs610604 mutation might be associated with the family history of psoriasis in Indians^22^. Orozco *et al*. found that the rs13207033 polymorphism conferred protection on RA, while the rs6920220 conferred susceptibility to RA^24^. Several studies showed that rs2230926 polymorphism was the susceptibility factor for SLE, RA and primary immune thrombocytopenia (ITP)^25–29^, but no association existed between rs2230926 and primary Sjogren’s syndrome (pSS)^30,31^. As for TNIP1 rs2233287, rs4958881 and rs3792783 polymorphisms, Bossini-Castillo *et al*. conducted a large independent replication study including 4389 SSc patients and 7611 healthy controls, which indicated that these three SNPs were significantly related to the risk of SSc^32^. In addition, significant association was observed between rs6889239 with Chinese SLE patients at allelic level^33^. Although those SNPs play a role in various autoimmune diseases, no statistical association was observed between the above eight SNPs with AS in this study, which may be partially due to the not large enough statistical powers in our study. However, the accurate effects on the pathogenesis of diseases remain difficult to pinpoint. Thus it still needs further association researches in independent cohort with larger samples to obtain explicit findings.

When it comes to TNFAIP3 rs10499194 polymorphism, we observed a significant association of AS with it. rs10499194 is a C to T base replacement located in the intergenic spacer upstream of TNFAIP3. Previous studies indicated significant relationships of rs10499194 with plenty of immune-related disorders. Graham *et al*. confirmed that rs10499184 was positively associated with SLE risk in a GWAS study^34^. A German case–control cohort study got a conclusion that rs10499194 variants increased the risk of multiple sclerosis (MS)^35^. At the same time, the mutation of rs10499194 play a protective role in some autoimmune diseases. In a GWAS, Plenge *et al*. reported that the minor allele at rs10499194 was associated with protection against RA^36^. Prahalad *et al*. found strong association of rs10499194 variants with a reduced juvenile idiopathic arthritis (JIA) risk after correction^37^, and their results confirmed the protective TNFAIP3 haplotypes associated with JIA. A recent research also reported that the T allele and CT genotype at rs10499194 decreased the risk of ITP^38^. Li *et al*. also observed that ITP individuals with rs10499194 CT genotype expressed higher levels of A20 mRNA than the CC genotype carriers, which further evidenced that the protective effect of rs10499194 mutation on ITP^38^. Furthermore, in our study, case–control regressions based on the reconstruction of TNFAIP3 haplotype block also disclosed a significant association with AS. The results showed a novel protective haplotype (rs13207033^A^- rs10499194^T^) associated with AS, suggesting that the existence of TNFAIP3 gene polymorphisms was an additional susceptibility related factor in the Chinese population with AS. In the analysis of single gene locus, rs13207033 polymorphism was not associated with AS predisposition, while rs13207033 came into play in the haplotype analysis, which might attribute to the combined effect of the two loci. To a certain extent, haplotype analysis is essential for better understanding how multiple SNPs in a gene contribute to the disease susceptibility, serving the purpose of illuminating pathogenic mechanisms of diseases.

In addition, we found that the wGRS was statistically different between AS patients and healthy controls. Additionally, the wGRS in HLA-B27 positive patients was different with that in HLA-B27 negative patients, indicating the different genetic background in TNFAIP3 between HLA-B27 positive and negative AS patients. We further evaluate the utility of wGRS to estimate multiple common variations in AS. The ROC curve analysis obtained a statistically significant result. But actually, only using wGRS of the five SNPs in TNFAIP3 gene was hard to distinguish AS individuals from healthy controls due to the modest sensitivity and specificity, which indicated that besides these polymorphisms, many other complex factors jointly contribute to the development of AS. Jung *et al*. developed a risk-scoring model for AS based on a combination of HLA-B27, SNP, and copy number variant markers, and found that the model could identify individuals at high risk for AS before major symptoms appear^39^. In a study including 11,366 RA cases and 15,489 healthy controls, even with all known genetic susceptibility variants to RA, prediction performance remains modest for the general population^40^. Hence, in the future, more susceptibility factors related to autoimmune diseases should be included into the model to obtain a better predictive effect.

Several limitations in our study should be considered. First, although our select criteria were quite strict, all participants were from one hospital, thus the selection bias was inevitable. Second, the nine gene loci we tested were obtained based on pervious reported loci related to other autoimmune disorders. This approach of gene selection cannot cover all susceptible loci, which may give rise to the selection bias. Third, after Bonferroni correction, the associations were no longer statistically significant, so the results should be interpreted with caution. However, this method has been criticized as too conservative, especially when plenty of tests are performed, because it may increase the risk of false negatives^41^. Furthermore, some scholars have stated that multiple comparison corrections should be forcefully considered for validation studies rather than exploratory studies^42,43^. Then, a replicated study focusing on the association of these SNPs with AS risk was not carried out. Next, geographical differences between Southern and Northern populations were not taken into consideration. Lastly, although the association of rs10499194 polymorphism and AS was observed and the functional annotation suggested a potential function of rs10499194 mutation, the exact biological effects of this SNP on AS development are not shown by functional experiments.

In summary, our study demonstrated that rs10499194 T allele and CT genotype in TNFAIP3 gene were associated with a reduced risk for AS. rs10499194 polymorphism decreased the risk of male, aged ≥29, BASFI <4, BASDAI <4 and HLA-B27 positive AS patients. In addition, the rs13207033^A^- rs10499194^T^ haplotype was associated with the decreased susceptibility to AS. We constructed the wGRS with the five SNPs in rs10499194, and observed that wGRS was different between AS patients and healthy controls and between HLA-B27 positive and negative patients. The functional annotation suggested a potential function of rs10499194 mutation. In the future, more case–control researches with comprehensive resequencing or SNP functional analysis are required to verify these preliminary findings.

## Materials and Methods

### Study subjects

In the present study, a total of 667 AS patients from the Department of Rheumatology and Immunology, the First Affiliated Hospital of Anhui Medical University (Hefei, China) were consecutively enrolled from March 2011 to September 2017. All patients were diagnosed by the skilled rheumatologist based on the modified 1984 New York Criteria and without any other immune-related diseases. In addition, we included 667 unrelated healthy controls from the physical examination center of the same hospital. All controls were healthy volunteers without any immune diseases or such family histories. The cases and controls were both Chinese Han populations who living in Anhui area in Eastern China. The healthy controls were comparable for gender and age to individual AS patients using propensity score matching. This study was conducted with the approval of the Ethics Committee of Anhui Medical University and informed consent was obtained from all individual participants included in the study. All procedures of this study conformed to the principles of the Declaration of Helsinki. All methods were conducted in accordance with the relevant guidelines and regulations.

### Demographic and clinical data collection

Demographic and clinical characteristics including gender, age, BMI, disease course, Age at presentation, HLA-B27 status, serum levels of erythrocyte sedimentation rate (ESR), C-reactive protein (CRP) and white blood cell (WBC) were obtained from self-administered questionnaires and medical records of AS patients. Disease activity of AS subjects was assessed by the Bath Ankylosing Spondylitis Disease Activity Index (BASDAI), and functional ability was evaluated by the Bath Ankylosing Spondylitis Functional Index (BASFI). The scores of both BASDAI and BASFI range from 0 to 10, and higher scores indicate the more severe disease activity or functional impairment.

### SNPs selection and genotyping

The tag SNPs (tSNPs) were selected based on linkage disequilibrium (LD) from the HapMap Data Rel 28 PhaseII + III, August10, using NCBI B36 assembly, dpSNP b126 and the Haploview 4.2 software. The SNPs were included if their minor allele frequency (MAF) was ≥ 5% among Han Chinese in Beijing (CHB). At last, Five SNPs in TNFAIP3 gene (rs610604, rs10499194, rs13207033, rs2230926 and rs6920220) and four SNPs in TNIP1 gene (rs2233287, rs3792783, rs4958881 and rs6889239) were chosen. Genomic DNA was extracted from peripheral venous blood of AS patients and healthy controls using a QIAGEN kit (QIAGEN, Hilden, Germany) based on the manufacturer’s instructions, and stored at −20 °C before the detection of genotyping. The genotyping of all SNPs was conducted using the improved Multiplex Ligase Detection Reaction (iMLDR) Assay technology (Shanghai Genesky Bio- Tech Co, Ltd.; www. geneskies. com). The primers are listed in Supplementary Table S1. Raw data were analyzed using GeneMapper 4.1 (Applied Biosystems, USA).

### Statistical analysis

Statistical analyses were conducted using SPSS version 23.0 (SPSS Inc., Chicago, IL, USA). Continuous data with normal distribution are given as mean ± standard deviation (SD), otherwise, data displayed as median (interquartile range, IQR), and categorical data were presented as absolute numbers and percentages. The comparison of normal distributed data was using *t* test, and data with skewed distribution were compared using Mann-Whitney *U* test. The distributions of allele frequencies, genotype and genetic models in patients and controls were compared using Chi-square or Fisher’s exact test. Odds ratios (ORs) with 95% confidence intervals (95% CIs) were calculated. Hardy–Weinberg equilibrium (HWE) was assessed in healthy controls by the Chi-square test. Linkage disequilibrium (LD) and haplotype analysis were implemented using Haploview 4.2 software. The weighted genetic risk score (wGRS) was computed for each individual. For this, firstly, each SNP were coded as 0, 1 or 2 according to the number of risk allele; then each SNP weighted using the natural logarithm of its OR; finally, all were summed and obtained the wGRS. The receiver-operating characteristic (ROC) curve and the area under the curve (AUC) were applied to evaluate the predictive value of wGRS. The gene-gene interaction was analyzed by multifactor dimensionality reduction (MDR; http://sourceforge.net/projects/mdr/). Statistical power was calculated by Quanto (http://hydra.usc.edu/gxe) based on additive model. Statistical significance was set at *P* < 0.05 (2 tails). While for multiple tests by Bonferroni correction, the corrected *P*-value < 0.05 was considered as statistically significant.

### Functional annotation

To further evaluate the potential functional features of significant SNPs, as well as SNPs in strong LD (*r*^2^ > 0.8) with these variants among East Asian population, genomic features were annotated using HaploRegv4.1^44^. Regulatory features, including several histone modification sites (H3K27ac, H3K4me1 and H3K4me3), of primary monocytes and T cells from peripheral blood and a lymphoblastoid cell line GM12878, were retrieved from the Roadmap Epigenomics Consortium (ROADMAP) and the Encyclopedia of DNA Elements (ENCODE). SNPs were mapped to regulatory elements utilizing UCSC genome browser^45^. After that, Phenotype-Informed Noncoding Element Scoring (PINES), a newly devised non-coding variants prediction tool, was used to assess the overall potentially functional variants because of its ability of good prediction for variants in non-coding regions (http://genetics.bwh.harvard.edu/pines/index.html). PINES provided a *P* value for each variant that involved in the evaluation. The smaller *P* value indicates the more potential functions that the variant might have.

### Ethical approval and consent to participate

All procedures performed in studies involving human participants were in accordance with the ethical standards of the institutional and/or national research committee and with the 1964 Helsinki declaration and its later amendments or comparable ethical standards.

## Supplementary information

supplementary files

## Data Availability

The datasets generated and analyzed during the current study are available from the corresponding author on reasonable request.
