# Supplementary material for: TNFAIP3 genetic polymorphisms reduce ankylosing spondylitis risk in Eastern Chinese Han population
Source: Sci Rep. 2019 Jul 15;9:10209. doi: 10.1038/s41598-019-46647-1 (PMC6629655; doi:10.1038/s41598-019-46647-1)
Supplement: Supplementary file 1 — supplementary files [file 41598_2019_46647_MOESM1_ESM.pdf]

Title: TNFAIP3 genetic polymorphisms reduce ankylosing spondylitis risk in Eastern Chinese Han population

Author list: Jiajia Yang[a,b,1], Xingxing Hu[a,b,1], Meng Wu[a,b], Yubo Ma[a,b], Xu Zhang[a,b], Mengya Chen[a,b], Yaping Yuan[a,b], Renfang Han[a,b], Rui Liu[a,b], Shiyang Guan[a,b], Jixiang Deng[a,b], Shanshan Xu[a,b], Xing Gao[a,b], Shengqian Xu[c], Zongwen Shuai[c], Shanqun Jiang[d], Shihe Guan[e], Liwen Chen[e], Faming Pan [a,b,*]

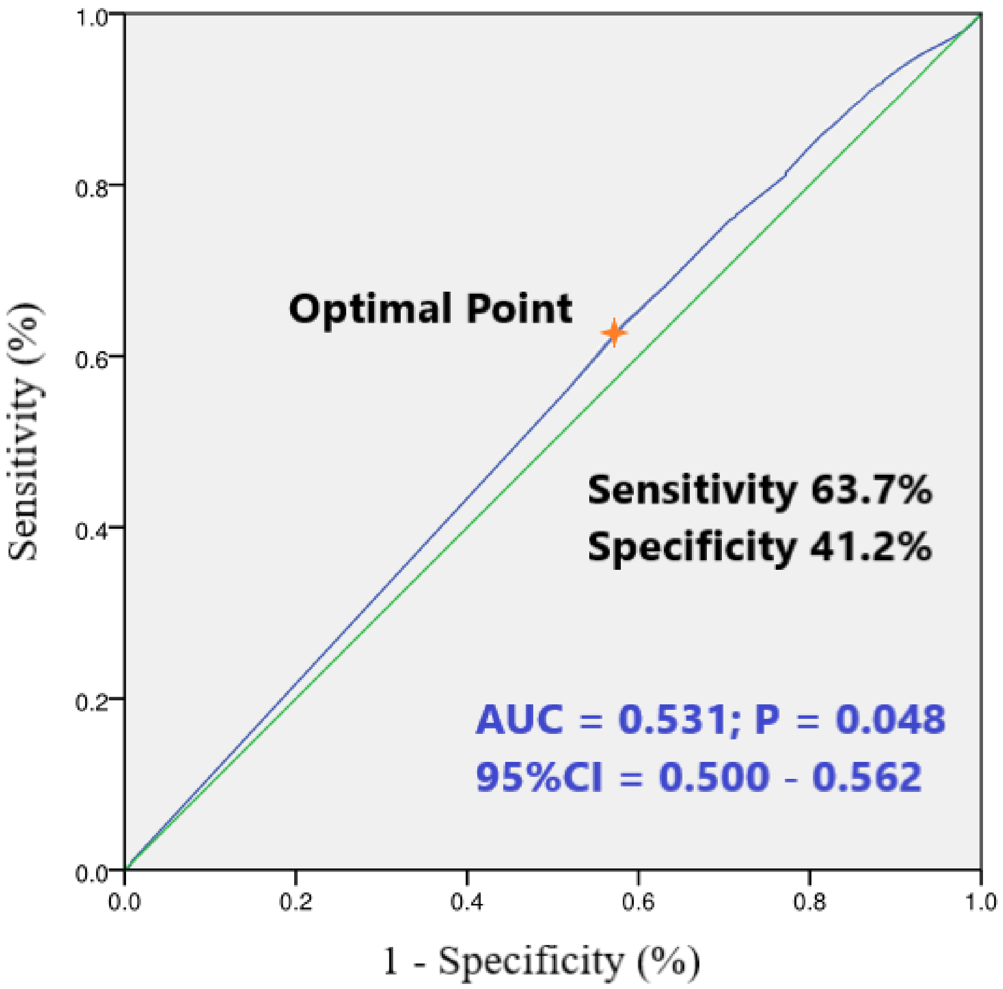

Supplementary Figure S1 Receiver operating characteristic (ROC) curve constructed using the weighted genetic risk score (wGRS).

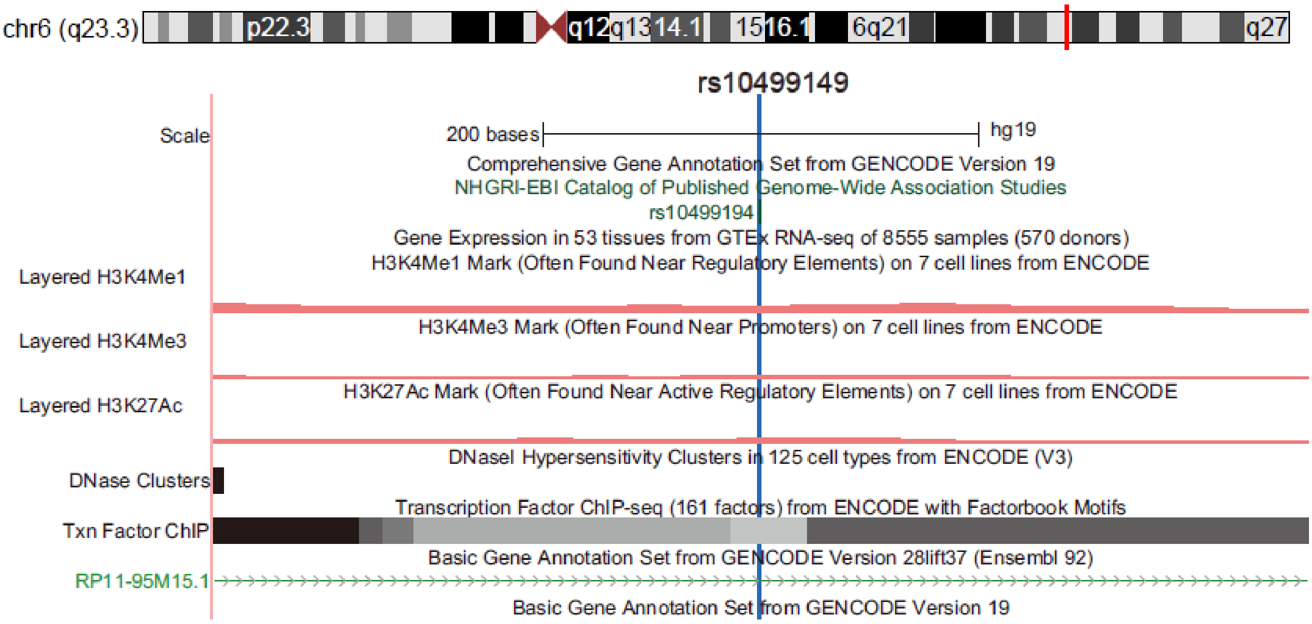

Supplementary Figure S2 Functional annotation of rs10499149 in lymphoblastoid cell line GM12878.

Supplementary Table S1 SNPs and PCR primers for TNFAIP3 and TNIP1 genes genotyping

SNPs	chromosome position	Allele	PCR primer
rs610604	138199417	T/G	F: ACACTTTTCACGCAACATGATCTG
			R: TGCAGGCCTGTGATGAAGAGATM
rs10499194	138002637	C/T	F:AACCCTTTTGTTCAAAAAGTAGTCATGTT
			R:AAATAGAGACAATATTGATTTTCTTCAAATGTGRTY
rs13207033	137965418	G/A	F:AAATCTGTAGAGGAGTTAAACAACACTGGA
			R: TGGGAAAAAAAAATGTCAAAGGGAAACTR
rs2230926	138196066	T/G	F: CAGCACGCTCAAGGAAACAGA
			R: TTGGTACTGAGGAAGGCGCTCTK
rs6920220	138006504	G/A	F: TTTTAGTGGAGAAGTTACCTGCTAACAGATG
			R: GCATATGCCATTGTTCTACAGAACCATARCY
rs2233287	150440097	G/A	F: ATGTGAGATGATATGTAACGGTCCCT
			R: TGTCGAGAATCCTGCTTTGTGATGTY
rs4958881	150450236	T/C	F: GCCATGATATCACATTGCAGACA
			R: CACCCAAGGATGAAAGGAAGTGACAY
rs3792783	150455732	A/G	F: GGGAAGTTCTTGATGATTTTCCCA
			R: GCGGCCACCATGACCATGGY
rs6889239	150457771	C/T	F: TGCAGTGTTCTTGATGCACAGG
			R: GACTCCTGTGCCCAGTGACR

TNFAIP3, tumor necrosis factor-α induced protein 3; TNIP1, TNFAIP3-interacting protein 1; SNPs, single nucleotide polymorphisms; PCR, polymerase chain reaction; F, forward; R, reverse; M,A/C; R,A/G; Y,C/T; K,G/T

Supplementary Table S2 Various models of inheritance of TNFAIP3 gene polymorphisms

SNPs	Genotype	AS (n, %)	HC (n,%)	OR(95%CI)	*P*	P_C
rs610604						
	TT	553 (82.9)	553 (82.9)	(Reference)		
	GT	108 (16.2)	106 (15.9)	1.019 (0.760─1.365)	0.900	NS
	GG	6 (0.9)	8 (1.2)	0.750 (0.259─2.176)	0.595	NS
	GT+GG	114 (17.1)	114 (17.1)	1.000 (0.752─1.330)	1.000	NS
	TT+GT	661 (99.1)	659 (98.8)	(Reference)		
	GG	6 (0.9)	8 (1.2)	0.748 (0.258─2.167)	0.591	NS
rs10499194						
	CC	617 (92.5)	588 (88.2)	(Reference)		
	CT	50 (7.5)	79 (11.8)	**0.603 (0.416─0.875)**	**0.007**	**NS**
	TT	0	0	NA	NA	NS
	CT+TT	50 (7.5)	79 (11.8)	**0.603 (0.416─0.875)**	**0.007**	**NS**
	CC+CT	667 (100)	667 (100)	(Reference)		
	TT	0	0	NA	NA	NS
rs13207033						
	GG	544 (81.6)	517 (77.5)	(Reference)		
	GA	113 (16.9)	139 (20.8)	0.773 (0.586─1.018)	0.066	NS
	AA	10 (1.5)	11 (1.7)	0.864 (0.364─2.052)	0.740	NS
	GA+AA	123 (18.4)	150 (22.5)	0.779 (0.597─1.018)	0.067	NS
	GG+GA	657 (98.5)	656 (98.3)	(Reference)		
	AA	10 (1.5)	11 (1.7)	0.908 (0.383─2.152)	0.826	NS
rs2230926						
	TT	621 (93.1)	614 (92.1)	(Reference)		
	GT	46 (6.9)	51 (7.6)	0.892 (0.590─1.349)	0.587	NS
	GG	0	2 (0.3)	1.003 (0.999─1.008)	0.155	NS
	GT+GG	46 (6.9)	53 (7.9)	0.858 (0.569─1.294)	0.465	NS
	TT+GT	667 (100)	665 (99.7)	(Reference)		
	GG	0	2 (0.3)	1.003 (0.999─1.007)	0.157	NS
rs6920220						
	GG	660 (99.0)	662 (99.3)	(Reference)		
	GA	7 (1.0)	5 (0.7)	1.404 (0.443─4.447)	0.562	NS
	AA	0	0	NA	NA	NS
	GA+AA	7 (1.0)	5 (0.7)	1.404 (0.443─4.447)	0.562	NS
	GG+GA	667 (100)	667 (100)	(Reference)		
	AA	0	0	NA	NA	NS

AS, ankylosing spondylitis; HC, healthy controls; SNPs, single nucleotide polymorphisms; OR, odds ratio; CI, confidence interval; TNFAIP3, tumor necrosis factor-α induced protein 3; NA, not available; NS, not significant; *Pc* , Bonferroni corrected *P*-value.

Supplementary Table S3 Various models of inheritance of TNIP1 gene polymorphisms

SNPs	Genotype	AS (n, %)	HC (n,%)	OR(95%CI)	*P*	P_C
rs2233287						
	GG	662 (99.3)	667 (100.0)	(Reference)		
	GA	5 (0.7)	0	0.993 (0.986─0.999)	0.073	NS
	AA	0	0	NA	NA	NS
	GA+AA	5	0	0.993 (0.986─0.999)	0.073	NS
	GG+GA	667 (100)	667 (100)	(Reference)		
	AA	0	0	NA	NA	NS
rs4958881						
	TT	563 (84.4)	560 (84.0)	(Reference)		
	CT	99 (14.8)	100 (15.0)	0.985 (0.728─1.331)	0.920	NS
	CC	5 (0.8)	7 (1.0)	0.710 (0.224─2.252)	0.560	NS
	CT+CC	104 (15.6)	107 (16.0)	0.967 (0.720─1.297)	0.822	NS
	TT+CT	662 (99.2)	660 (99.0)	(Reference)		
	CC	5 (0.8)	7 (1.0)	0.712 (0.225─2.255)	0.773	NS
rs3792783						
	AA	392 (58.8)	398 (59.7)	(Reference)		
	GA	246 (36.9)	234 (35.1)	1.067 (0.851─1.339)	0.573	NS
	GG	29 (4.3)	35 (5.2)	0.841 (0.504─1.403)	0.507	NS
	GA+GG	275 (41.2)	269 (40.3)	1.038 (0.834─1.291)	0.738	NS
	AA+GA	638 (95.7)	632 (94.8)	(Reference)		
	GG	29 (4.3)	35 (5.2)	0.821 (0.496─1.359)	0.442	NS
rs6889239						
	CC	382 (57.3)	375 (56.2)	(Reference)		
	CT	242 (36.3)	250 (37.5)	0.950 (0.757─1.192)	0.660	NS
	TT	43 (6.4)	42 (6.3)	1.005 (0.642─1.574)	0.982	NS
	CT+TT	285 (42.7)	292 (43.8)	0.958 (0.772─1.190)	0.699	NS
	CC+CT	624 (93.6)	625 (93.7)	(Reference)		
	TT	43 (6.4)	42 (6.3)	1.025 (0.661─1.591)	0.911	NS

AS, ankylosing spondylitis; HC, healthy controls; SNPs, single nucleotide polymorphisms; OR, odds ratio; CI, confidence interval; TNIP1, TNFAIP3-interacting protein 1; NA, not available; NS, not significant; *Pc* , Bonferroni corrected *P*-value.

Supplementary Table S4 MDR models of the interaction between TNFAIP3 and TNIP1 gene SNPs

Models	Training balance accuracy	Testing balance accuracy	Cross-validation consistency	*P*
rs10499194	0.5229	0.5037	6/10	0.8975
rs10499194, rs2230926	0.5301	0.4768	2/10	0.5583
rs13207033, rs610604, rs6889239	0.5420	0.4948	4/10	0.9009

MDR, multifactor dimensionality reduction; TNFAIP3, tumor necrosis factor-α induced protein 3; TNIP1, TNFAIP3-interacting protein 1.
